# The effect of digital reminiscence therapy on people with dementia: a pilot randomized controlled trial

**DOI:** 10.1186/s12877-020-01563-2

**Published:** 2020-05-06

**Authors:** SeolHwa Moon, Kyongok Park

**Affiliations:** 1 College of Nursing, Hanyang University, 222 Wangsimni-ro, Sungdong-gu, Seoul, 04763 Korea; 2 Department of Nursing, Gangneung-Wonju National University, 150 Namwon-ro, Heungeop-myeon, Wonju-si, Gangwon-do 26403 Korea

**Keywords:** Dementia, Depression, Mobile applications, Memory, Randomized controlled trial

## Abstract

**Background:**

Reminiscence therapy (RT) can improve various dysfunctions in people with dementia (PWD), but it may not be a cost-effective intervention. Digital RT allows multiple users to participate in a therapy simultaneously. Moreover, digital RT offers convenience, such as for uploading personal materials and presenting individual triggers of personal memories. This pilot study aimed to evaluate the effect of digital RT through a comparison with conventional RT and to develop a strategy for designing larger RCTs.

**Methods:**

An Android application and digital content were developed for digital RT. Overall, 49 PWD enrolled in nine daycare centers in Korea met the inclusion criteria. Eight sessions of digital RT in an intervention group (*n* = 25) and storytelling in a control group (*n* = 24) with no digital materials were performed over 4 weeks from February to June 2019. Cognition, depression, behavioral and psychological symptoms of dementia (BPSD), and engagement were measured as the primary outcomes to evaluate the effect of digital RT. All outcomes except for engagement were evaluated at baseline before the intervention (T0), post-intervention (T1), and 4 weeks after the intervention (T2). Engagement was measured at the first and last intervention sessions. Differences in cognition, depression and BPSD between groups and across time points (T0, T1, and T2) were analyzed by repeated measures ANOVA. Differences in engagement between the groups and time points (the first and last sessions) were analyzed by independent t-tests. This study adhered to the CONSORT guidelines.

**Results:**

Depression (F = 7.62, *p =* .001, partial η^2^ = .17) was significantly decreased at T1 and T2, and engagement (t = − 2.71, *p* = .011) was significantly increased at the last session in the digital RT group compared to the control group. However, cognition (F = 0.13, *p* = .821) and BPSD (F = 0.67, *p* = .485) were not significantly different between groups and time points.

**Conclusions:**

Digital RT proved an innovative approach to manage PWD and will thus help PWD achieve a better mood and have more opportunities to engage in social interactions.

**Trial registration:**

KCT0003446 in the Clinical Research Information Service. Registered 24 January 2019, https://cris.nih.go.kr/cris/search/search_result_st01.jsp?seq=14391

## Background

Dementia is an irreversible condition presenting not only cognitive dysfunction, depression, and behavioral and psychological symptoms of dementia (BPSD) but also dysfunction in activities of daily living (ADL). Globally, dementia is the fifth leading cause of death and the most burdensome disease [1]. Dementia affected 50 million people across the globe in 2018 [2] and 9.8% of Koreans over the age of 65 in 2019 [3]. Care costs for dementia patients exceed 10 billion USD, and dementia is one of the costliest diseases in Korea [4]. The percentage of those over 65 with dementia is expected to increase to 15.1% by 2050 [4], and care costs will increase accordingly.

Recently, some countries have developed policies to promote the early detection and prevention of dementia and to improve treatment and care for people with dementia (PWD) because of social burdens such as increasing care costs and the number of PWD [5]. With the rise in social interest in caring for PWD, a variety of nonpharmacological interventions have been receiving popular attention. According to the guidelines for dementia care, nonpharmacological interventions should be implemented before considering the use of medication [6]. Among the various nonpharmacological interventions, reminiscence therapy (RT) has been used in a number of long-term care facilities [7, 8].

RT helps people recall their past experiences and adds meaning to their lives [9]. RT can improve cognitive function and quality of life for PWD, allowing them to decrease or better manage their depression and other dementia symptoms [7, 8, 10, 11]. The psychosocial improvement induced by RT can benefit PWD as well as caregivers [12]. Conventional RT, such as storytelling, has been performed and requires memory triggers, including household items or objects related to past events. In performing conventional RT, the person leading the RT must be trained, and items functioning as memory triggers must be prepared; therefore, RT may not be cost effective [13, 14].

To improve the accessibility and usability of RT, researchers have implemented therapy with advanced information and communication technologies (ICTs), such as digital RT, digital life stories, and networked RT [14–17]. Digital RT using ICTs is a practical method to support RT delivery using multiple engaging media and allowing for multiple users; it can use webcams, photos, interactions with computer graphics, and personalized videos [14–16]. This digital content may include more or stronger visual and auditory stimuli for enhanced PWD engagement [17]. Moreover, digital RT in the form of mobile applications (apps) is convenient because the apps do not require specialized space or equipment, and users can easily upload personal materials to stimulate their specific memories. Regarding convenience, users can adjust the screen size for mobile devices such as phones and tablets, which can increase users’ attention and ease of use.

Although digital RT is convenient and effective for PWD, there have been only a few trials to verify the effectiveness of digital RT compared to conventional RT, and the results of the trials were not consistent [17–21]. Therefore, the objective of this pilot study was to evaluate the effect of digital RT through a comparison to storytelling as a conventional RT approach for PWD. The objective of this pilot study was also to test this intervention with a small sample of PWD to establish a foundation for larger RCTs.

## Methods

### Development of a mobile application for reminiscence therapy and usability test

**Fig. 1:**
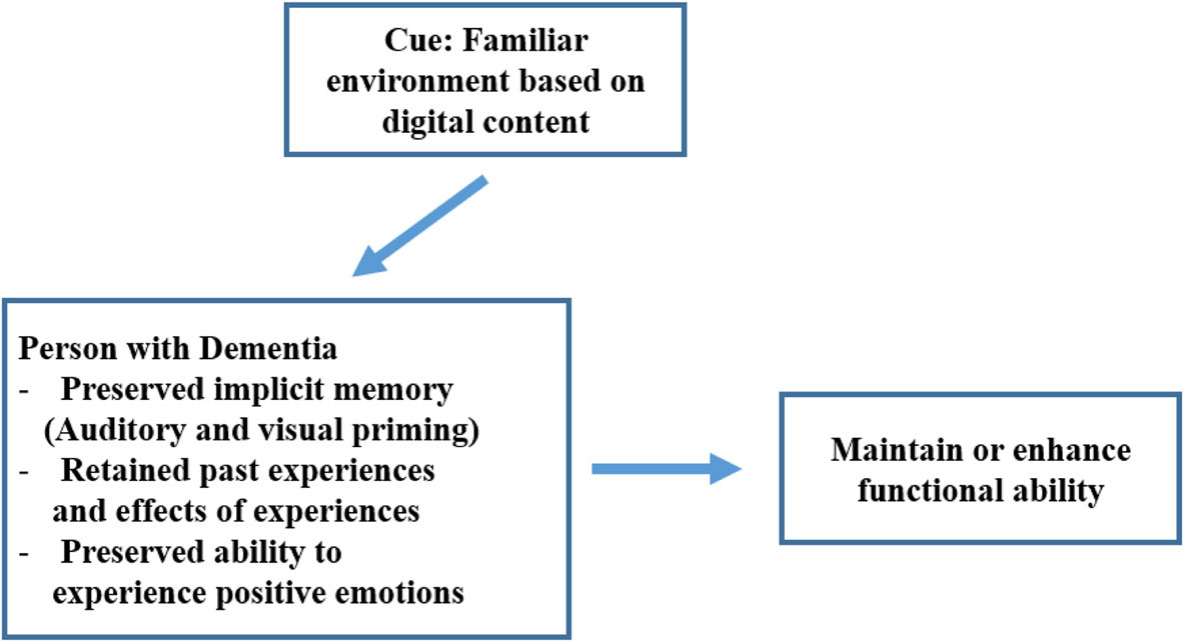
Conceptual Framework of This Study

The digital RT for PWD in this study is based on a model of familiarity among elders with dementia [22]. The conceptual framework of this study is shown in Fig. 1. The model of familiarity among elders with dementia proposes that being in familiar environments surrounded by familiar objects can make people feel more comfortable, both consciously and unconsciously. Familiarity can be a memory trigger in PWD through auditory and visual priming; eventually, PWD in familiar environments will be able to maintain or enhance their functional ability [23–25]. To create the content of the digital RT for PWD, materials for a familiar digital environment for PWD were searched on public websites such as NAVER, Google, and YouTube. To avoid legal or copyright issues, the materials retrieved were only items that were marked as noncommercial under Creative Commons License regulations and, if necessary, created videos were paid for.

**Fig. 2:**
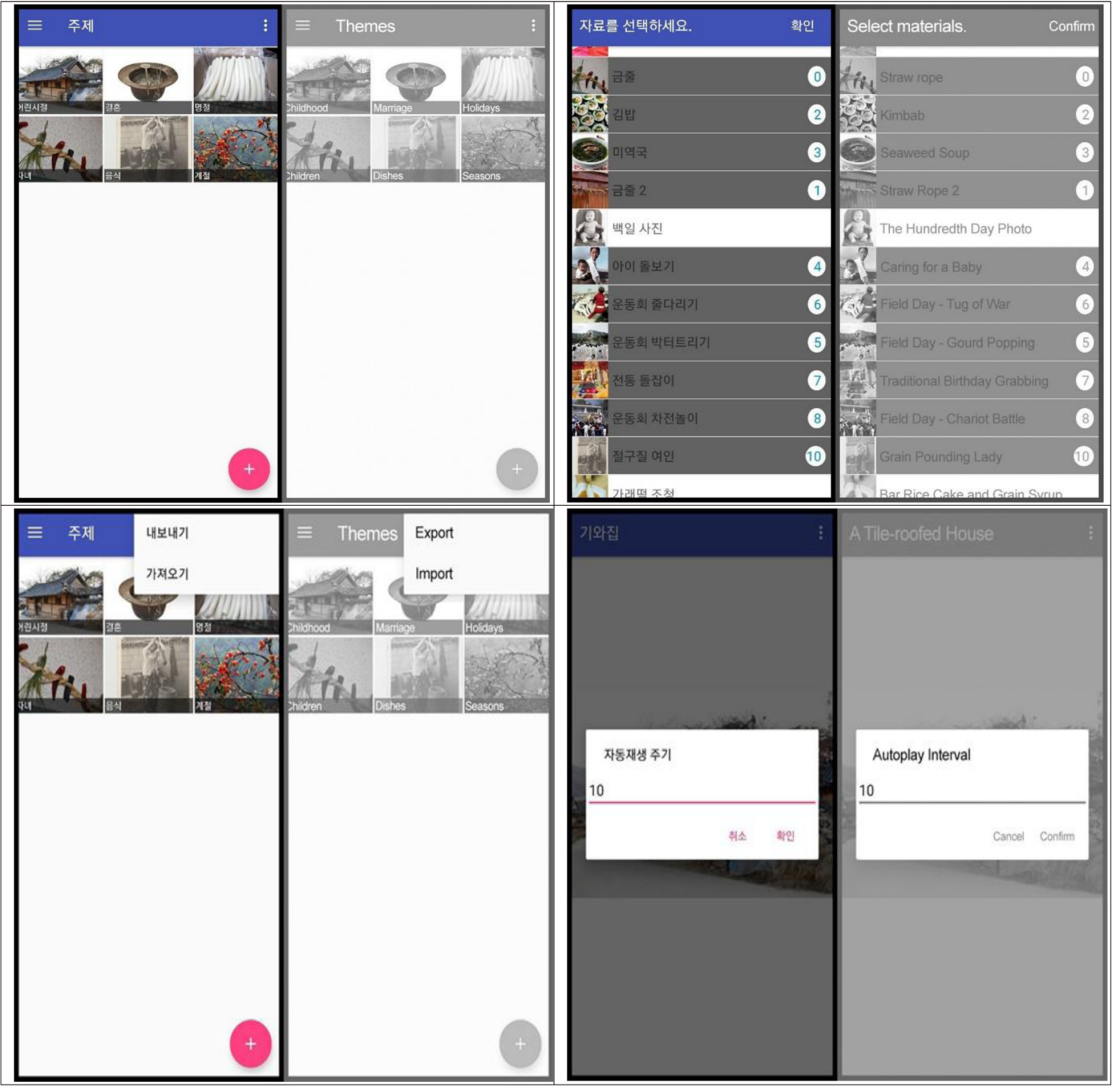
Functions of the Smartphone Application for Reminiscence Therapy

The mobile RT app was developed from June 2018 to November 2018. It was built on the Android platform because this is the most popular platform in Korea. The app allows users to immediately conduct digital RT sessions onsite with pre-downloaded media and to add personalized material. The smartphone application for the digital RT is shown in Fig. 2. Video and image files stored on the device or YouTube URLs can be added to the app. The app also allows users to play their files in auto-play mode and to select the auto-play interval. YouTube videos were uploaded to the app with an embedded YouTube player app. Users could export all or selected materials to other devices (Fig. 2).

To collect personal information and materials, researchers also interviewed PWD and their family members about their past experiences. Researchers conducted a telephone interview with caregivers of PWD, and each interview lasted approximately 20 to 40 min. Interviews were carried out with family members such as sons, daughters-in-law, and daughters who were familiar with the PWD and knew them well. During the interview, information was collected by asking open-ended questions about past experiences with the PWD. The interview topics were as follows: childhood, marriage and youth, holidays, children and preferences (hobbies, activities, music, food, etc.).

During the personal interviews, researchers requested from participants’ family members personal materials such as photos of children, grandchildren, spouses, or family events such as weddings, birthdays, or family trips. After receiving the photos via mail, a researcher scanned the images and made a digital file and returned the materials to the family members. Through the interviews with the PWD and their family members, the researchers identified that participants had common cultural experiences of the twentieth century, and researchers chose themes and materials for each reminiscing session to elicit maximum sympathy and familiarity. Finally, six themes for the digital reminiscence sessions, namely, childhood, marriage, holidays, children, food, and seasons, were selected [13, 16, 26].

After developing the content and app for the mobile digital RT, a usability test was conducted with four PWD at daycare centers. The usability test participants also met the inclusion criteria of the current study. The usability test was conducted by the research staff in the program rooms of two daycare centers in February 2019 and lasted approximately 20–30 min. The app for the usability test included 52 photos and three videos. The usability test had a limitation in that participants’ responses were measured qualitatively. However, the researchers and staff in the daycare centers observed the usability test and offered comments on the composition or content of the digital RT and the app functions. The results of the usability test indicated that the resolution of the photos or videos, the size of screen, and the auto-play interval were acceptable for application in the program for PWD. The appropriate length of the program and the volume of the sound were revised because the potential participants may have decreased vision and/or hearing.

### Effectiveness test for digital reminiscence therapy

#### Study design and setting

This pilot study is a single blind randomized controlled trial. Parallel group randomization was conducted with an allocation ratio of 1:1.

This study adheres to the CONSORT guidelines. This pilot trial was registered with the Clinical Research Information Service (CRIS) (KCT0003446). The basic study information can be accessed at https://cris.nih.go.kr/cris/search/search_result_st01.jsp?seq=14391. The study was approved by the Institutional Review Board of Far East University, the corresponding author’s former affiliation (FEUIRB− 180,531-01-2).

#### Participants

Overall, 49 participants who met the inclusion criteria were enrolled in this research. The inclusion criteria for participants were as follows: 1) female ≥65 years old; 2) moderate dementia [27, 28] (Mini-Mental Status Examination 10–19); 3) no impairment of hearing or vision; 4) registered at the daycare center for more than a month. Participants were excluded if they changed medication during the intervention period and were diagnosed with a psychiatric disease, except for depression.

This pilot study included only females because meaningful memories based on familiarity and implicit memory are related to life habits or familiar skills and vary by gender [16, 22]. There are a few trials on reminiscence based on a single-gender sample [29, 30]. To evaluate the effect of digital RT and develop a strategy for larger RCTs, following convention, this pilot study included female participants.

This present study included only moderate (MMSE score 10–19) dementia [27, 28]. Thus, we excluded those with mild dementia (MMSE score ≥ 20) because mild dementia patients rarely register at daycare centers in Korea. We also excluded severe dementia (MMSE score ≤ 9) patients because implicit memory priming is less effective in severe stages with PWD [25].

The investigation and comparison of outcomes in the digital RT and conventional storytelling groups were performed from February to June 2019. Recruitment of participants from the daycare center began after registration approval from the CRIS in January 2019 and after obtaining permission from the staff at the daycare centers. First, two daycare centers were registered with the CRIS on Feb 18, 2019. The intervention began immediately after obtaining consent from all participants’ families to reduce the dropout rate. Participant dropout occurs frequently among older adults at daycare centers because of moves to other areas, discharge from the daycare center, new disease diagnoses, or changes in the main caregiver. Next, the remaining 4 daycare centers were registered with the CRIS on March 4, 2019. Finally, 3 daycare centers were registered with the CRIS on April 29, 2019.

#### Interventions

Digital RT was performed one-on-one for both the intervention and control groups from February to June. PWD in familiar environments not only feel more comfortable but are also able to maintain and improve their functional ability [23–25]. Since implicit memory is triggered by familiar visual and auditory stimuli, the present study evaluated these effects by providing a familiar environment using digital content. Participants belonging to the intervention group joined in the digital RT using a tablet PC with a much wider screen than a smart phone screen considering the participants’ decreased visual function. RT was conducted with a 5-min introduction, followed by a 20-min reminiscence and a 5-min wrap-up. The intervention was conducted by three registered nurses. The nurse interventionists leading the RT were trained in how to control the application on a tablet PC and how to lead the app-based RT with PWD. The intervention was conducted from 10 to 11:30 am or 2 to 4 pm for approximately 30 min for each participant in a separate program room at the daycare center. The same program was provided twice a week in eight sessions over 4 weeks. To realize an individual approach, the participant characteristics collected through the interviews with family members were shared with the interventionists before the intervention. The following characteristics were shared: people or events that the participants like or dislike or things that the participants like or dislike. The digital RT program included PWD’s preferred song and a personal photo. Information about the preferred song and audio files were provided to interventionists before the intervention. The information on group allocation was concealed prior to starting the intervention.

Storytelling without digital materials was also performed individually and face-to-face for PWD in the control group by the same nurse interventionists that guided the PWD in the digital group. In a separate program room, the nurse interventionists talked with the participants belonging to the storytelling group without digital materials about the same six themes focused on in the digital RT group. Storytelling was also performed for 30 min in eight sessions over 4 weeks, as in the digital group. Participants belonging to the storytelling group were also encouraged to sing a song, reflecting the participants’ preference without an audio file.

#### Outcome measurement

Outcomes were measured by researchers to minimize bias in measurement. Two researchers received training regarding the same measurement methods and thresholds and were blinded to the group allocation and intervention. Cognition, depression, BPSD, and engagement were measured as primary outcomes. All outcomes except for engagement were evaluated at baseline before the intervention (T0), post-intervention (T1), and 4 weeks after the intervention (T2). Engagement was measured during the first session in the first week of the intervention and at the last session in the 4th week of the intervention.

The main variables are as follows.

***Cognition*** The MMSE-DS was used to assess cognitive function [27]. The scale consists of 19 items. Thirty points is the highest possible total score. A higher score means better cognitive function. The Cronbach’s alpha coefficient of internal consistency for the MMSE-DS is 0.83 [27]. The Kuder-Richardson formula 20 value for the MMSE-DS was 0.84 in this study.

***Depression*** The Cornell Scale for Depression in Dementia (CSDD) was developed as an observational scale to evaluate depression in PWD [31]. The Korean version of the CSDD [32] was used by the nursing staff at each daycare center. The K-CSDD consists of 19 items, with a total score ranging from 0 to 38. A higher score indicates more depressive symptoms. The Cronbach’s α of the K-CSDD was 0.88 in a previous study [32] and was 0.76 at baseline in this study.

***Behavioral and psychological symptoms of dementia (BPSD)*** A neuropsychiatric inventory (NPI) was developed by Cummings and colleagues in 1994 [33]. The Korean version of the neuropsychiatric inventory (K-NPI) was used to measure BPSD [34]. The K-NPI consists of 12 behavior domains. The frequency and severity of each domain of the K-NPI observed by the caregivers was assessed. The total score ranges from 0 to 144, and higher scores indicate severe BPSD. The Cronbach’s α of the K-NPI was 0.85 in a previous study [34] and was 0.78 at baseline in this study.

***Engagement*** The Engagement of a Person with Dementia Scale (EPWDS) was used to measure the degree of engagement during the RT [35]. The EPWDS consists of five areas of engagement: affective, visual, verbal, behavioral, and social. Each area is divided into two opposite items indicating positive and negative engagement. The ten items are rated on a 5-point Likert scale, and the total score ranges from 10 to 50. Before using the EPWDS in this study, the English version of the EPWDS was translated to Korean and back-translated to English by four bilingual experts in gerontology. The content equivalence or semantic equivalence of the back-translated version of the EPWDS was assessed with a 3-point Likert scale (3 = exactly the same meaning in both versions, 2 = almost the same meaning in both versions, and 1 = different meaning in each version). Seven of the 10 items showed 100% agreement among the four bilingual experts regarding content equivalence or semantic equivalence. Three items presenting 80% agreement were revised. The translated version of the EPWDS revised for more acceptable equivalence was applied in the present study. The EPWDS was used after training raters to improve the inter-rater reliability. The Cronbach’s α of the original version of the EPWDS was 0.94 [35]; it was 0.74 in the current study.

#### Sample size

The sample size was calculated with G*power 3.1 and was based on a systematic review of the effect of RT [7, 8]. Two systematic reviews were used to confirm the effect size, and an effect size of .27 was applied in this study. Forty-one participants were needed to achieve a power of 0.95 at three measurement times (α = 0.05) [36]. However, this study included 49 participants to account for a 20% dropout rate in older adults with dementia [37].

#### Randomization and blinding

Simple randomization was determined using a computer generated table of random numbers. Participants were allocated to the intervention and control groups with an equal allocation ratio using SPSS Statistics software 21.0 (IBM, Armonk, New York, USA).

The allocation sequence was also concealed from the participants, interventionists, and researchers measuring the outcomes until the participants were assigned to interventions and started the intervention. Performance bias is evitable in nonpharmacological interventions [7, 13]. Enrollment of participants was performed by the researchers responsible for communicating with the daycare centers and for interviewing the participants’ family members. Allocation to each intervention group was performed by the research assistant, who was able to operate the computer program according to the researchers’ instructions. Outcome measurement was also performed by two doctoral students, and the allocation information was concealed from the doctoral students. The two trained interventionists were registered nurses, and the allocation information was also concealed from the interventionists until the intervention started.

#### Analysis

SPSS Statistics software 21.0 (IBM, Armonk, New York, USA) was used for statistical analysis of the data. The Shapiro-Wilk test, skewness, and kurtosis were used to evaluate the normal distribution of the variables [38]. Data transformation [39] was conducted for variables such as “number of medications taken” and “depression at T1” which did not show a normal distribution. General characteristics were summarized by descriptive analysis. To confirm the homogeneity of the two groups at baseline, independent t-tests and x^2^-tests were conducted. After confirming the homogeneity of the variables between the groups, we analyzed the differences between the two groups and at the three measurement times (T0, T1, and T2) in cognition, depression and BPSD using repeated measures ANOVA. Engagement was measured by how participants participated in the intervention and interacted with the interventionist during the RT program. Independent t-tests were conducted to determine the mean difference between the 1st and last sessions of the intervention.

## Results

**Fig. 3:**
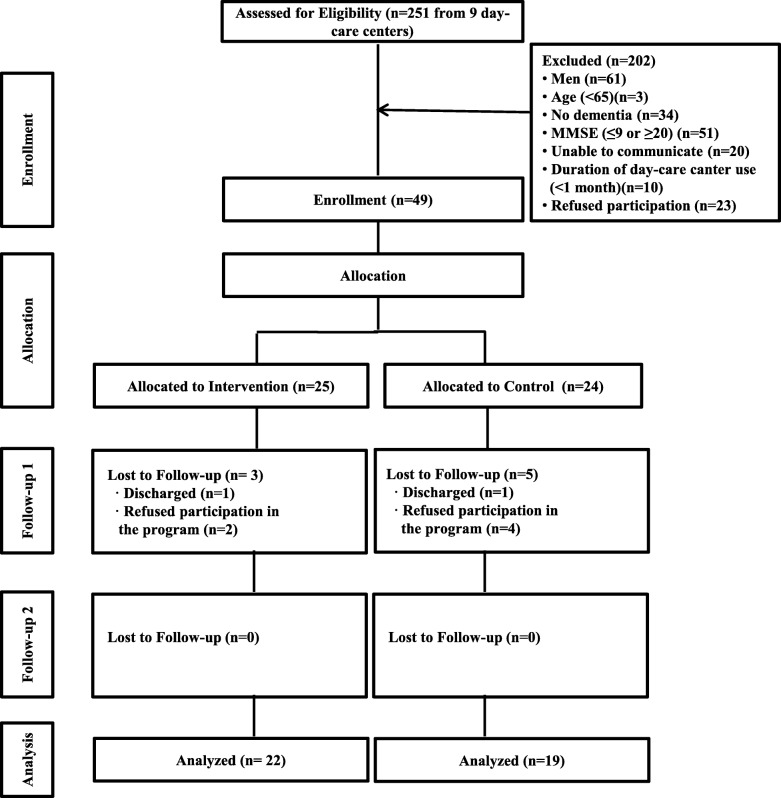
Study Flow Chart

The study flow diagram is shown in Fig. 3. Overall, 251 candidates were assessed to determine whether they met the inclusion criteria. At the beginning of the enrolment process, 43 candidates from two daycare centers were screened. After that, five PWD were enrolled in the study and allocated to the intervention or control group using a computer program; each group had the same allocation ratio. After enrolling 5 PWD, the researcher contacted the potential participants and their family caregivers and explained the purpose of the study to them. Verbal consent was obtained and recorded during a telephone interview with the family caregivers. The next 88 candidates from four daycare centers were screened, and then 120 additional candidates from three daycare centers were screened. It took several months to recruit participants and obtain consent from their family members because there are few large-scale daycare centers in the two local study areas in Korea, and only a few PWD in each daycare center met the inclusion criteria of this study. Ultimately, 251 candidates from nine daycare centers in two local areas in Korea were screened, 49 participants were enrolled, and 24 and 25 participants were assigned to the intervention and control group, respectively, from February to June 2019. However, data from 22 participants in the intervention group and 19 participants in the control group were analyzed because some participants were discharged from their facility or refused to participate in the program (Fig. 3).

The mean age of the participants was 83.46 ± 6.06 years. The age of the participants ranged from 68 to 96 years. The general characteristics of the participants are shown in Table 1. The homogeneity test revealed no significant difference between the two groups in general characteristics and main variables (Table 1). The effect of digital RT in the two groups is shown in Table 2. Depression (F = 7.62, *p =* .001, partial η^2^ = .17) was significantly decreased in the intervention group compared to the control group at T1 and T2. A post hoc test showed significant differences at T0 and T1 (*p* = .023) and T0 and T2 (*p* = .012). Cognition (F = 0.13, *p* = .821) and BPSD (F = 0.67, *p* = .485) were not significantly different between groups and time points. The change in engagement during the RT intervention is shown in Table 2. The mean value of engagement in the last session was 3.79 ± 3.82 higher in the digital RT group but -0.86 ± 6.01 lower in the control group than in the first session. The mean difference was statistically significant (t = − 2.71, *p* = .011).

**Table 1:** Demographic Characteristics of the Sample and Homogeneity of Groups at Baseline (*N* = 41)

	Cont (*n* = 19)	Exp (*n* = 22)	t / x^2^/z	*p*
	n (%) or M ± SD	n (%) or M ± SD		
Age (years)	84.05 ± 6.23	82.96 ± 6.01	0.57^a^	.570
Education (years)	4.00 ± 4.00	2.91 ± 3.29	0.958 ^a^	.344
Diagnosis				
Alzheimer disease	9 (47.4)	8 (36.3)	0.163^b^	.922
Vascular dementia	3 (15.8)	4 (18.2)		
Other	7 (36.8)	10 (45.5)		
Number of chronic disease	1.63 ± 0.76	1.96 ± 0.95	-1.54^c^	.124
Number of medications taken*	2.27 ± 1.31	2.83 ± 2.14	−1.24	.223
Cognition	15.11 ± 3.62	14.73 ± 4.04	0.32 ^a^	.384
Depression	4.42 ± 3.52	6.14 ± 4.78	−1.29 ^a^	.204
BPDS	17.95 ± 14.76	16.24 ± 10.10	0.43 ^a^	.669

*Cont* control group, *Exp* experimental group; * Transformed data; ^a^ t value; ^b^ x^2^ value; ^c^ z score; *BPSD* behavioral and psychological symptoms of dementia

**Table 2:** Comparison of the Effect between Digital RT and Traditional RT Groups (*N* = 41)

Variables	Time	Cont (n = 19)	Exp (n = 22)	Source	F/t	*p*
		M ± SD	M ± SD			
Cognition^a^	T0	15.11 ± 3.61	14.50 ± 4.16	Group	0.22	.643
	T1	16.39 ± 3.65	16.15 ± 3.73	Time	4.66	.022
	T2	16.50 ± 4.27	15.75 ± 4.38	G*T	0.13	.821
Depression^a^	T0	4.42 ± 3.52	6.14 ± 4.77	Group	0.20	.654
	T1*	4.25 ± 3.12	3.20 ± 2.94	Time	6.84	.002
	T2	4.68 ± 2.21	2.82 ± 3.04	G*T	7.62	.001
BPDS^a^	T0	18.78 ± 14.72	16.24 ± 10.10	Group	1.02	.319
	T1	16.72 ± 14.10	13.14 ± 11.57	Time	6.46	.005
	T2	16.22 ± 14.09	11.19 ± 7.30	G*T	0.67	.485
Engagement^b^	1st session	47.67 ± 2.35	45.05 ± 3.59			
	last session	46.50 ± 6.36	48.79 ± 1.62			
	Mean difference	−0.86 ± 6.01	3.79 ± 3.82		−2.71^c^	.011

*Cont* control group, *Exp* experimental group, *T0* before intervention, *T1* Post-intervention, *T2* 4 weeks after intervention; ^a^ repeated measures- ANOVA; ^b^ independent t-test; ^c^ t value, *BPSD* behavioral and psychological symptoms of dementia; * Transformed data, *Group* main effect of group, *Time* main effect of time, G*T group x time interaction effect, *Mean difference* last session- 1st session

## Discussion

Systematic reviews examining the effects of RT in PWD have reported that RT is effective for cognitive, emotional, or behavioral manifestations of dementia [7, 8, 11]. Even though RT has been shown to be effective for various symptoms of PWD, conventional RT requires collecting items related to past events and training staff to administer the RT. Therefore, conventional RT may not be a cost-effective intervention [13, 14]. Digital RT is an innovative method, although the process of developing the application can be expensive. Once made, the application can be repeatedly and easily used without requiring real objects as cues for recalling past events [14]. Moreover, digital RT can offer numerous types of engaging multimedia materials, including photos, audio, and videos. Compared to conventional RT, such as storytelling, these kinds of multimedia materials can provide PWD with many stimuli. Eventually, PWD can gain rich opportunities to participate in social interactions through these stimuli and take ownership of the conversation [14].

This study was designed to administer individual RT reflecting individuals’ desires and preferences. In one trial, the authors recommended that the life review process should be individualized because people may choose to spend more time than anticipated focusing on specific life stages [40]. The authors of one systematic review also suggested that individualized therapy reduced depression in PWD more effectively than did a group approach [7]. Although it can be challenging to hold interviews with PWD and their family members to collect individualized data, digital RT using a mobile app makes it easy to upload and add digital individual data such as family photos, favorite pictures, and music. Therefore, digital RT would be more convenient for users.

The present study evaluated the effect of digital RT on community-dwelling PWD. The results showed that depression was significantly lower in the digital RT group than the storytelling group after the intervention. Depressed mood is a common occurrence in PWD and affects quality of life. Even though the underlying mechanisms of depression are unknown, endogenous brain defense activity such as brain reserve or neuroplasticity may have an effect on depression and the aging process [41], and nonpharmacological interventions such as RT may also have a positive effect on depression [6–8]. Several studies have reported that depression was decreased in PWD after RT using digital media [17, 18]. One trial study reported on an eight-week intervention with Memory Box that involved computer files such as favorite music, photographs, and movies. The results showed that the PWD exhibited decreased symptoms of depression and anxiety after the intervention [18]. Subramaniam and Woods (2016) conducted a pilot study to evaluate the effect of RT using digital life storybooks [17]. The authors reported that digital life storybooks were useful tools for participants with mild to moderate dementia to improve depressive symptoms and evoke positive emotions and memories.

Participants’ engagement in the present study was significantly higher in the digital RT group. Previous studies found that the use of technologies such as digital content for older adults with dementia improved meaningful engagement [19, 20]. McCauley and colleagues also suggested that RT using digital materials reflecting personal preference can evoke an individual’s interest and actively induce participation [21]. Therefore, digital content reflecting familiarity and multisensorial stimuli could prompt the recall of enjoyable implicit memories in participants and lead to more engagement in social interaction. Based on the results of this study and previous studies, personalized digital RT can decrease depressed mood and increase social engagement for PWD.

The MMSE-DS score representing cognitive function increased at T1 in the digital RT group. However, the MMSE-DS score decreased at T2. One systematic review reported that there was an effect of RT on cognition for PWD, but there was little or no difference at longer-term follow-up [8]. This report was consistent with our results. Another systematic review reported that the effect on cognition of RT for PWD was not consistent across the studies included [7]. A previous study on the effect of digital RT on PWD reported improvement in cognitive functions, but the sample of the RCT was small [42]. Therefore, there are limitations to generalizing the results regarding cognition. It is necessary to conduct further studies to identify the effect on cognition of digital RT.

BPSD was decreased at T1 and T2. However, there were no significant differences between groups. Several studied have reported results different from those reported here. One systematic review of RT for PWD reported that there were effects on BPSD [7], but another study reported no clear effect on BPSD [8]. Studies have reported that digital RT for PWD is effective for agitated or apathetic behavior [18, 42]. These results are not consistent. The present study included only female participants and had a small sample size. Therefore, further studies with large sample sizes and including both genders are required.

This study had several limitations. The participants in this study were all women. This study is based on “the model using familiarity and implicit memory in PWD” [22]. Implicit memory encompasses non-declarative memory and procedural memory, such as habits or skills. These life habits or familiar skills vary by gender. During the process of creating digital content for RT, the research team realized that the meaningful memory events for women and men were very different. Women were more likely to recall family events such as a wedding, caring for a child, and making food, whereas men were more likely to recall political events, military service, and their job. Therefore, further study is suggested so that digital RT content for men can be developed and large-sample RCTs including male participants can be performed to generalize the effects of digital RT.

It was not clearly proven whether digital RT had an effect on the cognition or BPSD of PWD. Based on the results, cognitive function did not increase at T2. Another study also reported that the effectiveness of RT at follow-up was not clear [8]. Therefore, we recommend that when planning further RCTs, measurement should be performed only at baseline and post-intervention, not follow-up, and the analysis method should involve independent t-tests with a larger sample.

This study did not identify the cost effectiveness of digital RT even though it is easy and convenient to use for PWD or caregivers. Therefore, studies on the cost effectiveness of digital RT are required.

## Conclusions

This study was conducted to evaluate the effect of digital RT and to develop a strategy for designing larger RCTs. Depression was decreased and engagement was improved in the digital RT group compared to the control group after the intervention. With digital RT, individual materials can easily be added when conducting individual therapy. As a consequence, individual digital RT could provide personalized care reflecting individuals’ desires and preferences and eventually contribute to conducting person-centered care.

## Data Availability

The datasets used and/or analyzed during the current study are available from the corresponding author upon reasonable request.

## Abbreviations

RT: Reminiscence therapy
PWD: People with dementia
BPSD: Behavioral and psychological symptoms of dementia
ADL: Activities of daily living
ICTs: Information and communication technologies
app: application
MMSE-DS: Mini-mental status examination for dementia screening
CSDD: Cornell scale for depression in dementia
NPI: Neuropsychiatric inventory
EPWDS: Engagement of a person with dementia scale
